# Luteinizing hormone-releasing hormone in prostate cancer treatment: from hypothalamic discovery to clinical translation

**DOI:** 10.1093/jncics/pkag075

**Published:** 2026-07-13

**Authors:** Samuel Benisti, Fred Saad, Guila Delouya, Daniel Taussky

**Affiliations:** Faculty of Medicine, University of Montreal, Montreal, QC, Canada; Division of Urology, Department of Surgery, Centre Hospitalier de l’Université de Montréal (CHUM), Montreal, QC, Canada; Department of Radiation Oncology, Centre hospitalier de l’Université de Montréal, Montreal, QC, Canada; Department of Radiation Oncology, Centre hospitalier de l’Université de Montréal, Montreal, QC, Canada

## Abstract

**Introduction and Objectives:**

This project elucidates the pivotal scientific discoveries that culminated in the isolation of luteinizing hormone-releasing hormone (LHRH). It details the contributions of scientists Andrew Schally and Roger Guillemin, whose competitive biochemical research led to the Nobel Prize in 1977.

**Materials and Methods:**

This project entails a comprehensive review of the pivotal scientific developments that culminated in the discovery of LHRH, examined through the historical lens. The sources included peer-reviewed articles, Nobel lectures, and oral history transcripts.

**Results:**

Harris first showed the anterior pituitary’s regulation by the hypothalamus in 1937, establishing the principles for Schally and Guillemin. Schally and Guillemin independently isolated TRF in 1969, requiring 160 000 porcine hypothalami for purification. Both then pursued LHRH, isolating it independently in 1971 using 240 000 porcine brains and developing advanced biochemical techniques. These methods have enabled LHRH analog synthesis for prostate cancer research. In 1971, Schally demonstrated that LHRH could suppress gonadotropin secretion. Synthetic LHRH infusion in monkeys showed LHRH’s ability to desensitize pituitary gonadotropin-releasing hormone receptors. This discovery enabled the use of LHRH analog treatments for hormone-sensitive conditions. Schally conducted the first clinical trials in Mexico in 1972 and organized the first LHRH agonist study for prostate cancer, which was published in 1982.

**Conclusions:**

The discovery of LHRH represents a landmark convergence of evolving physiological concepts, rigorous biochemical innovation, and impactful clinical translation, leading to the Nobel Prize. The rivalry between Schally and Guillemin led to foundational discoveries that transformed the management of androgen-dependent malignancies.

## Introduction

The secretory regulation of the anterior pituitary by the hypothalamus has long been a subject of intense scientific investigation, evolving from early neural-endocrine models to modern neurohumoral paradigms. Initial physiological and anatomical studies suggested hypothalamic control through neural pathways; however, definitive biochemical proof of hypothalamic releasing factors remained elusive until the mid-20th century.

Luteinizing hormone-releasing hormone (LHRH), more commonly referred to as gonadotropin-releasing hormone (GnRH), is a hypothalamic decapeptide that regulates the secretion of pituitary gonadotropins. The structural elucidation of LHRH in 1971 culminated over a decade of intense investigation by Andrew Schally and Roger Guillemin, who independently isolated and characterized the decapeptide from porcine and ovine hypothalamic tissues, respectively.^1^^,^^2^

Before them, Harris in 1937 proposed a neurohumoral hypothesis that the hypothalamus controls anterior pituitary function through humoral factors transported via the hypothalamic-pituitary portal circulation rather than direct neural connections.^1^ Early purification efforts in the 1960s by Harris and colleagues demonstrated that LHRH was a basic peptide with a molecular weight of 1200-2500, after a 200 000-fold purification effort from sheep hypothalamic tissue.^3^ The race between Schally and Guillemin for the discovery of LHRH accelerated the discovery and the early human clinical trials that established the feasibility of medical castration in prostate cancer. The 1971 discovery by both the groups, within a few days and independently of each other, revealed identical decapeptide structures across species, enabling rapid synthesis and clinical testing.^1^^,^^4^ This work earned both investigators the 1977 Nobel Prize in Physiology or Medicine and transformed reproductive endocrinology.^2^

**Figure 1.**
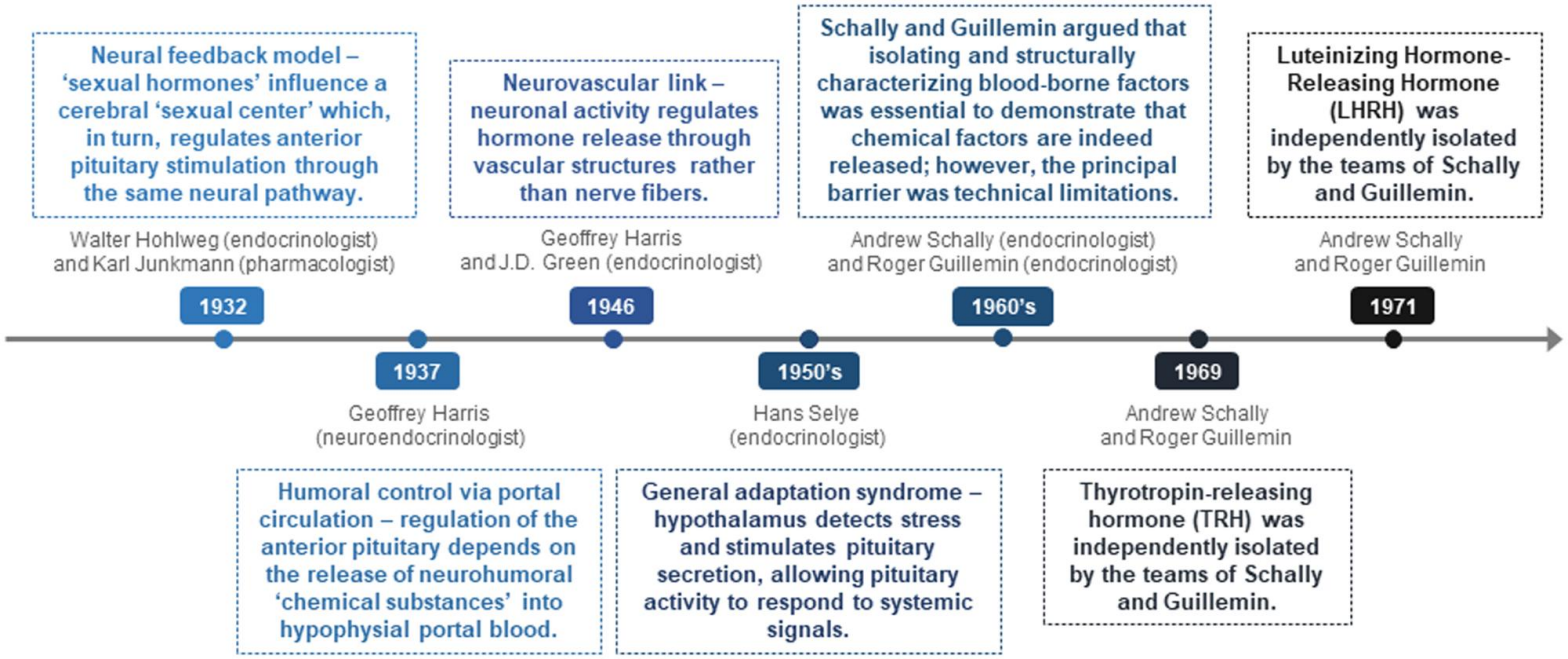
Conceptual, biochemical, and clinical evolution leading to LHRH-based therapy.

The first prostate cancer patient was treated with a GnRH agonist in 1979 at the Université Laval in Quebec City in Canada, leading to rapid worldwide adoption as an alternative to surgical castration and high-dose estrogen therapy.^5^ Early phase 2 trials in 1982 by Tolis et al. in Montreal, Canada, demonstrated that daily subcutaneous administration of the synthetic LHRH analog [D-Trp6]LHRH or intranasal HOE 766 (buserelin), another synthetic LHRH analog, achieved 75% testosterone suppression by 3 weeks, accompanied by decreased acid phosphatase levels, clinical improvement in prostatism, and relief of bone pain in metastatic patients.^6^ Trachtenberg’s 1983 study of [D-Leu-6]LHRH nonapeptide ethylamide in 9 patients with stage D2 disease confirmed that significant testosterone suppression and tumor stabilization could be achieved without major adverse effects.^7^ In this narrative review, we outline the historical context, biochemical challenges, and translational significance of LHRH, integrating physiological theory, molecular endocrinology, and the eventual clinical applications of this work. Figure 1 summarizes how the discovery of LHRH emerged from converging conceptual, biochemical, and translational advances that ultimately enabled modern androgen-deprivation therapy.

## Methods

A structured historical-analytic approach was used, integrating original scientific reports, contemporaneous commentaries, and subsequent historical analyses to reconstruct the conceptual, biochemical, and therapeutic development of LHRH analog therapy.

Primary sources were identified using targeted searches of PubMed and Web of Science. The search terms included “luteinizing hormone-releasing hormone,” “gonadotropin-releasing hormone,” “hypothalamic releasing factors,” “TRH isolation,” “Schally,” “Guillemin,” and “prostate cancer hormonal therapy.” The reference lists of key publications were also reviewed to identify further relevant sources.

The included materials comprised (1) original experimental studies describing hypothalamic hormone isolation and sequencing, (2) historical accounts of the scientific competition surrounding thyrotropin-releasing hormone (TRH) and LHRH, and (3) translational and clinical studies evaluating LHRH analogs in androgen-dependent malignancies. Reports unrelated to mammalian systems or lacking primary data or historical relevance were excluded from the study. We also reviewed transcripts of an interview with Guillemin that provides first-person accounts of laboratory decisions, interpersonal dynamics, and methodological improvizations unavailable in published papers.

The analysis was conducted in 3 stages: conceptual foundations of hypothalamic regulation, biochemical isolation followed by definitive structural characterization, and translational development linking continuous LHRH administration to medical castration in prostate cancer. The findings were synthesized thematically to trace the progression from physiological inference to molecular identification and eventual clinical application.

## Results

### Early theories of anterior pituitary secretion

By the early 20th century, physiologists began to suspect that the hypothalamus exerted regulatory control over anterior pituitary.^8^^,^^9^ Anatomical research had demonstrated neural continuity between the hypothalamus and pituitary; however, the mechanism behind this influence—direct neural transmission or blood-borne mediation—remained uncertain.^8^ In the absence of identifiable mediators, hypothalamic control was inferred rather than experimentally demonstrated.

Before hypothalamic releasing factors could be isolated or chemically conceived, anterior pituitary regulation was frequently interpreted using hybrid neural-endocrine models rather than through a strictly blood-borne mediator. In 1932, Hohlweg and Junkmann^10^ analyzed gonadal-pituitary relationships within a feedback framework in which circulating “sexual hormones” influenced a central nervous “sexual center,” which, in turn, regulated anterior pituitary activity using neural impulses.^10^ In this model, diminished gonadal hormone production increases the stimulatory drive from the central nervous system to the anterior pituitary gland, whereas elevated gonadal hormone levels dampen pituitary stimulation through the same neural pathway.^10^

This was not framed as a metaphor for the future. The authors explicitly discussed the search for anatomical “secretory pathways” within the vegetative (autonomic) nervous system and considered sympathetic pathways as plausible mechanisms of pituitary modulation, while admitting the experimental difficulty in proving such pathways existed.^10^ In this conceptual framework, endocrine feedback was real, but its execution was imagined as neurogenic: circulating hormones altered activity within a central neural regulator, and that regulator then acted upon the pituitary gland.

Given the methodological limitations of the period, this reasoning was physiologically coherent. Endocrine feedback can be detected experimentally; however, no discrete hypothalamic mediators had been isolated. Therefore, a neural intermediary offers an intelligible bridge between peripheral hormonal signals and pituitary output.^11^

Significant research contributions originated from the work of Hans Selye at l’Université de Montréal in Canada. He was a physiologist, endocrinologist, and medical researcher who launched the field of stress physiology. His formulation of the general adaptation syndrome placed the pituitary gland at the center of systemic stress responses, stimulating interest in the mechanisms by which the hypothalamus regulates pituitary secretion. Although only Guillemin trained directly with Selye in Montreal, Schally’s contemporaneous work at McGill in Montreal placed him within the same vibrant endocrine research landscape. While Harris provided the anatomical and physiological framework for hypothalamic control, Selye’s research on stress popularized the idea that pituitary function responds to systemic signals. His work had created a generation of investigators.^12^ Harris argued that anterior pituitary regulation depended on humoral factors transmitted through the hypophyseal portal circulation.^1^ In doing so, he repositioned the hypothalamus as an endocrine organ rather than merely a neural structure, linking anatomical observations with physiological functions. However, the proposal remained incomplete without biochemical proof. As Guillemin later recalled, Harris’ lectures in Montreal “created a new theory: the pituitary gland as subservient to the hypothalamus,” reshaping Guillemin’s scientific trajectory.^13^

However, by the early 1960s, expectations within endocrine physiology had shifted. In their 1964 synthesis, Schally, Bowers, and Locke argued that definitive confirmation of hypothalamic neurohumoral regulation would require isolation and structural characterization of the responsible factors, paralleling the resolution of posterior pituitary peptides, once their chemistry was defined.^8^ Indirect bioassay responses and plausible neurogenic mechanisms were no longer sufficient. If anterior pituitary control depends on blood-borne factors, these factors must be demonstrated as chemically identifiable entities.^8^

Thus, prior to the isolation of TRH, the central debate in endocrine physiology was not simply “nerves vs blood,” but rather the level of evidence required to establish hypothalamic control of the pituitary gland. While neural models could explain observed feedback phenomena without invoking discrete chemical messengers, the emerging neurohumoral hypothesis requires demonstrating specific blood-borne releasing factors.^8^^,^^10^ As emphasized by Schally, Bowers, and Locke, definitive confirmation necessitated isolation, structural characterization, and evidence of secretion into the circulation. Until the chemical identification of these molecules was achieved, the hypothesis concerning the releasing factor was not confirmed.^9^ The principal barrier was not conceptual resistance but technical limitations. Hypothalamic extracts contain biologically active peptides in minute concentrations embedded inside complex tissue matrices, rendering them indistinguishable from contaminants using mid-century bioassays.^9^^,^^10^ Indeed, Guillemin noted that collecting hypothalamic fragments at slaughterhouses made him “recognize the immensity of the project.”^13^ The sensitivity required for purification and sequencing exceeds contemporary biochemical capability. Although physiological experiments had suggested gonadotropin-releasing activity, the absence of molecular identification had precluded definitive experimental modeling.^8^^,^^14^ Skepticism persisted within segments of the scientific community.^8^^,^^15^ Without structural identification, hypothalamic releasing factors remained difficult to distinguish from experimental artifacts.^8^^,^^15^ The distance between physiological inference and molecular proof began to shrink only with the isolation of TRH.^14^

### TRH as biochemical validation of the releasing-factor hypothesis

The isolation of TRH in 1969 provided the first biochemical confirmation of the releasing-factor hypothesis.^14^ After purification from porcine hypothalamic extracts and demonstration of biological activity, it became clear that structural definition was possible.^16^ The identification of TRH as a tripeptide (pyroglutamyl-histidyl-proline amide) showed that hypothalamic hormones are discrete chemical entities that can be sequenced and synthesized.^14^

The importance of TRH extended beyond its identification. Its isolation validated large-scale extraction, followed by bioassay-guided chromatographic fractionation.^14^^,^^16^ Independent confirmation of the TRH structure by competing laboratories dispelled skepticism.^14^^,^^15^ With TRH, what had long appeared technically implausible became demonstrably attainable.^14^ If 1 releasing factor could be isolated despite its scarcity, others might yield similar strategies.^14^ Therefore, attention had shifted toward the more complex target, LHRH.^14^^,^^15^

### The race for LHRH: structural identification and scientific competition

Luteinizing hormone-releasing hormone, the hypothesized regulator of gonadotropin secretion, proved to be substantially more elusive. Present in lower concentrations than TRH and biologically more difficult to assay reliably,^8^ early physiological studies demonstrated LH release in response to crude hypothalamic extracts without identifying a defined molecular species.^6^^,^^14^

By the late 1960s, the pursuit of LHRH had evolved into a high-intensity scientific race between Schally’s laboratory at Tulane University and Guillemin’s group at Baylor College of Medicine.^15^^,^^17^

Both recognized that definitive structural identification would represent the final molecular validation of Harris’ portal hypothesis.^4^ Schally’s program relied heavily on unprecedented large-scale tissue procurement and iterative bioassay-guided purification, whereas Guillemin’s laboratory pursued parallel strategies supported by advances in peptide chemistry techniques.^15^^,^^17^ Competition accelerated methodological refinement, and experimental findings were closely guarded as each group approached structural confirmation.^15^^,^^17^

The scale of this undertaking was extraordinary. Approximately 160 000 porcine hypothalami were processed to obtain microgram quantities of the purified material.^2^^,^^14^ Schally later recalled that “I personally did the isolations.… Only a person such as myself, with unshakable faith in hypothalamic hormones, would endure the many laborious steps of purification.”^18^

Sequential gel filtration, ion-exchange chromatography, countercurrent distribution, and electrophoretic techniques were employed, with each fraction undergoing bioassay tracking.^11^^,^^14^ The instability of certain amino acids, including tryptophan residues, which are susceptible to degradation during purification, complicates sequencing efforts and requires meticulous methodological control.^14^ Minor losses of biological activity can obscure progress, and assay variability frequently delays definitive conclusions.^8^^,^^17^

**Figure 2.**
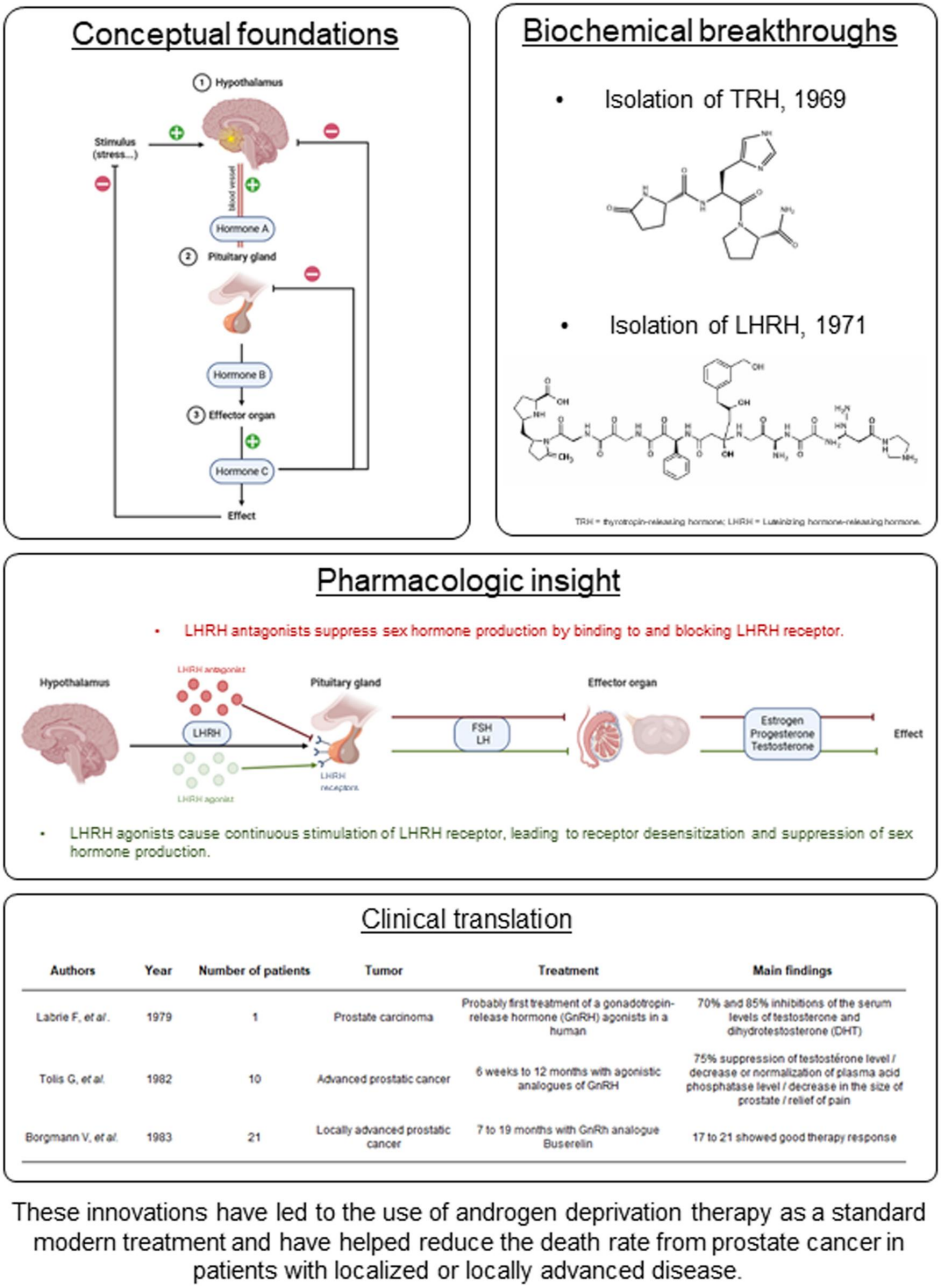
Timeline of key discoveries in hypothalamic-pituitary regulation (1932-1971).

Structural analysis ultimately revealed LHRH to be a decapeptide (pGlu-His-Trp-Ser-Tyr-Gly-Leu-Arg-Pro-Gly-NH_2_).^2^^,^^14^ Synthetic reproduction confirmed the biological activity and eliminated the possibility of nonspecific extract effects.^14^ The near-simultaneous publication of structural identification by the 2 laboratories underscored both the intensity and legitimacy of the race.^15^^,^^17^  Figure 2 presents a chronological overview of the major conceptual and experimental milestones that shaped the understanding of hypothalamic-pituitary regulation from 1932 to 1971.

In 1977, Schally and Guillemin shared the Nobel Prize in Physiology or Medicine for their discoveries concerning peptide hormone production in the brain.^15^^,^^17^^,^^19^ With LHRH structurally defined, the neurohumoral hypothesis articulated decades earlier had been translated into molecular certainty.^8^^,^^9^^,^^14^

### Physiological paradoxes and their relevance for therapy

Under physiological conditions, LHRH is secreted in a pulsatile manner, stimulating LH and FSH release.^14^ Initial investigations had largely focused on its stimulatory role.^14^ Later studies with synthetic analogs revealed an unexpected paradox: continuous administration produced an initial “flare” in gonadotropin secretion, followed by sustained suppression.^2^ This phenomenon reflects receptor desensitization and downregulation.^2^ Continuous exposure reduces LH secretion and suppresses testicular testosterone production.^2^

By exploiting receptor desensitization, LHRH agonists made reversible medical castration possible.^2^

### Translational applications in androgen-dependent malignancies

Continuous LHRH administration reproduces the endocrine effects of orchiectomy without surgical intervention.^13^ Preclinical studies confirmed the inhibition of androgen-dependent prostate tumor growth^14^ and early clinical investigation demonstrated sustained testosterone suppression and tumor regression in patients with advanced prostate cancer.^15^ Schally later described himself as an “endocrine oncologist,” underscoring the transition from hypothalamic hormonal research into cancer therapeutics.^18^

In 1979, Labrie was likely the first to describe the clinical utility of a GnRH agonists in a human at the Université Laval in Québec City.^5^ His team gave the agonist buserelin to a patient suffering from stage B prostate cancer intranasally that resulted in a 70% and 85% reduction in the serum levels of testosterone and dihydrotestosterone. In an article published in 2002,^20^ Schally revisited his initial clinical trials and stated that initial studies using natural LHRH were validated through experiments using synthetic LHRH in the 1970s. These trials conducted in Mexico with TRH and LHRH were significantly ahead (“by a wide margin”) of any other clinical research. The first significant clinical study illustrating the therapeutic potential of LHRH agonists in the treatment of prostate cancer was conducted in Montréal and published in PNAS in 1982,^6^ followed by a report in *The Prostate* in 1983.^21^

The study published in PNAS demonstrated that LHRH agonists effectively induce medical castration in male patients with prostate cancer. Additionally, complementary studies using rat models revealed that the suppression of testosterone through this method produces antitumor effects that are comparable to those achieved by surgical orchiectomy.

The initial clinical use of LHRH agonists in men with advanced prostate cancer attempted to confirm whether the experimentally observed endocrine paradox could be applied to oncologic therapy.^15^ In this early study of patients with advanced disease, continuous administration of a synthetic LHRH agonist resulted in the expected biphasic hormonal response: an initial increase in gonadotropin and testosterone levels, followed by a sustained decrease to castration levels.^15^ The transient flare phase, while biologically anticipated, highlights the difficulties inherent in applying receptor pharmacology to clinical practice.

By linking pituitary receptor desensitization to evident tumor regression, this trial established a link between molecular endocrinology and therapeutic oncology. An initial effort to isolate and characterize a hypothalamic peptide ultimately led to the development of a reversible endocrine intervention capable of modifying the clinical trajectory of androgen-dependent malignancies.^13^^,^^15^ Subsequent analyses have highlighted the therapeutic importance of hypothalamic hormones in oncology.^6^ Through pharmacologic manipulation of receptor signaling, what had formerly been understood primarily as a physiological regulator became a practical therapeutic tool, establishing LHRH analog therapy as an effective form of hormonal therapy.^6^^,^^13^^,^^15^

## Conclusion

The history of LHRH moved from neural-endocrine models of pituitary regulation to biochemical isolation to clinical application in prostate cancer. Early physiological frameworks could explain endocrine feedback without invoking discrete releasing substances, but by the 1960s, the standard for confirmation had shifted toward structural identification. Parallel efforts at Tulane University and Baylor College culminated in the near-simultaneous sequencing of LHRH and formal recognition of its significance.

The subsequent demonstration that continuous LHRH administration could suppress testosterone to castration levels in men with advanced prostate cancer established a direct therapeutic link between molecular endocrinology and oncology. In this sense, the discovery of LHRH was not only a biochemical achievement but also a convergence of conceptual evolution, experimental rigor, and translational success leading to the therapeutic foundation of advanced prostate cancer.

## Data Availability

The data supporting the findings of this narrative review were derived from publicly available sources, including peer-reviewed articles, Nobel lectures, and oral history transcripts. All primary sources used for this historical and analytical review were accessible through academic databases such as PubMed and Web of Science. No new experimental data were generated during this narrative review. For further details or specific data requests, please contact the corresponding authors.

## Use of AI

We used generative AI tools (Microsoft Copilot, OpenEvidence) to assist with literature summarization and the initial drafting of the Background and Methods sections. The AI output was used solely as a drafting aid. All factual claims, interpretations, and final wording were independently verified, edited, and approved by the authors of this study. No AI tool was used to generate the original data or analyses. We also used Paperpal for language refinement purposes. The authors retain full responsibility for the integrity and accuracy of this article.
